# Different sulfonylureas induce the apoptosis of proximal tubular epithelial cell differently via closing K_ATP_ channel

**DOI:** 10.1186/s10020-018-0042-5

**Published:** 2018-09-04

**Authors:** Rui Zhang, Xiaojun Zhou, Xue Shen, Tianyue Xie, Chunmei Xu, Zhiwei Zou, Jianjun Dong, Lin Liao

**Affiliations:** 1 Department of Endocrinology, Shandong Provincial Qianfoshan Hospital, Shandong University, Jinan, Shandong China; 2 Department of Endocrinology, Qilu Hospital of Shandong University, Jinan, Shandong China; 3 Division of Endocrinology, Department of Internal Medicine, Shandong University of Traditional Chinese Medicine, Jinan, Shandong China; 4 Department of Internal Medicine, Division of Endocrinology, Qilu Hospital of Shandong University, No. 107, Wenhuaxi Road, Jinan, Shandong China; 5 Department of Internal Medicine, Division of Endocrinology, Shandong Provincial Qianfoshan Hospital, Shandong University, No. 16766, Jingshi Road, Jinan, Shandong China

**Keywords:** Diabetes kidney disease, Proximal tubular epithelial cells, ATP-dependent potassium channel, Glibenclamide, Glimepiride, Gliclazide

## Abstract

**Background:**

Sulfonylureas (SUs) are widely prescribed for the treatment of type 2 diabetes (T2DM). Sulfonylurea receptors (SURs) are their main functional receptors. These receptors are also found in kidney, especially the tubular cells. However, the effects of SUs on renal proximal tubular epithelial cells (PTECs) were unclear.

**Methods:**

Three commonly used SUs were included in this study to investigate if different SUs have different effects on the apoptosis of PTECs. HK-2 cells were exposed to SUs for 24 h prior to exposure to 30 mM glucose, the apoptosis rate was evaluated by Annexin/PI flow cytometry. Bcl-2, Bax and the ratio of LC3II to LC3I were also studied by western blot in vitro. Diazoxide was used to evaluate the role of K_ATP_ channel in SUs-induced apoptosis of PTECs. A Student’s t-test was used to assess significance for data within two groups.

**Results:**

Treatment with glibenclamide aggravated the apoptosis of HK-2 cells in high-glucose, as indicated by a significant decrease in the expression of Bcl-2 and increase in Bax. Additionally, the decreased LC3II/LC3I reflects that the autophagy was inhibited by glibenclamide. Similar but less pronounced change was found in glimepiride group, however, nearly opposite effects were found in gliclazide group. Further, the effects of glibenclamide on apoptosis promotion and the decreased LC3II/LC3I were ameliorated obviously by treatment with 100uM diazoxide. The potential protection effect of gliclazide was also inhibited after opening the K_ATP_ channel.

**Conclusion:**

Our results suggest that, the effects of glibenclamide and glimepiride on PTECs apoptosis, especially the former, were achieved in part by closing the K_ATP_ channel. In contrast to glibenclamide and glimepiride, therapeutic concentrations of gliclazide showed an inhibitory effect on apoptosis of PTECs, which may have a benefit in the preservation of functional PTECs mass.

## Background

Sulfonylureas (SUs) is one of the most commonly prescribed class of drugs for treatment of type 2 diabetes mellitus (T2DM) (Tahrani et al. 2016). SUs binds to their receptors (sulfonylurea receptor, SUR), which are subunit of the ATP-dependent potassium (K_ATP_) channel, thus closing the K_ATP_ channel in pancreatic β-cells, resulting in insulin secretion and decreases blood glucose (Gribble & Reimann, 2003).

Diabetic kidney disease (DKD) is one of the major microvascular complications of diabetes (Zoja et al. 2015). Glomerulopathy associated with diffuse or nodular glomerulosclerosis was originally deemed as the main pathologic change (Ilatovskaya et al. 2015). Researchers have recently come to appreciate the key role played by proximal renal tubules in DKD (Wakino et al. 2015; De Nicola et al. 2014). Studies show that one-third of diabetic patients with microalbuminuria having no or minimal glomerular changes, only proximal tubular lesions (Singh et al. 2008). Tubulopathy, especially the apoptosis of proximal tubular epithelial cells (PTECs), is shown to play an important role in DKD, which occurs earlier than glomerulopathy (Magri & Fava, 2011; Tojo & Kinugasa, 2012; Barzilay et al. 2013).

The effects of SUs on DKD have been thought to be due to their indirect effects via their ability to decrease blood glucose (Giannico et al 2007). Few studies have examined whether SUs have direct effects on the kidney. SURs are present in a wide variety of extra-pancreatic tissues (Gribble & Reimann, 2003). Investigations indicated that SUR2, one common subtype of SUR, is located in PTECs (Zhou et al. 2008; Szamosfalvi et al. 2002). Hence, it is conceivable that SUs could act directly on the PTECs. As a K_ATP_ channel blocker, SUs close the K_ATP_ channels, leading to membrane depolarization and opening of voltage-operated Ca^2+^ channels, which further cause Ca^2+^ influx, and intracellular rise of Ca^2+^. This process may subsequently induce Ca^2+^-dependent-apoptosis (Efanova et al. 1998). Several studies have shown that the opening of the K_ATP_ channel is renoprotective (Shiraishi et al. 2014; Assad et al. 2009; Zhang et al. 2013). So, SUs might have detrimental effects on kidney, which is a concern in clinical practice.

The aim of our present study was to explore the effects of three widely prescribed SUs (glibenclamide, glimepiride and gliclazide) on the apoptosis of PTECs, an important progress in the development of DKD. To determine whether these SUs could be differentiated with regard to their effects on the apoptosis of PTECs and to analyze their possible underlying molecular mechanisms. Our results showed that glibenclamide and glimepiride, especial the former, promotes the apoptosis of PTECs through interacting with K_ATP_ channels. These pro-apoptosis effects of glibenclamide and glimepiride are mediated, at least in part, via downregulation of autophagy activity in renal tubular cells. To the contrary, gliclazide showed an inhibitory effect on apoptosis of PTECs.

## Methods

### Cell culture

Human proximal tubular epithelial cells (Ryan et al. 1994) (HK-2, American Type Cell Collection, Rockville, MD) were cultured in the RPMI 1640 medium containing 10% fetal bovine serum (Gibco, USA), 11.1 mM glucose, 100 U/ml penicillin and 100 μg/ml streptomycin(Sigma, St. Louis, MO) at 37 °C, 5% CO_2_ and 95% humidity. The culture medium was replaced with fresh medium every 2–3 days and expanded to new culture plates when the cell reach approximately 80% confluence.

### Antibodies, drugs and reagents

Antibodies in the study were from the following sources: anti-Bcl-2 and anti-Bax (polyclonal) from Proteintech Group(Chicago, IL), anti-LC3(Mono) from Cell Signaling Technology(Beverly, MA), anti-β-actin from Sigma(St. Louis, MO). All secondary antibodies (polyclonal) were from Jackson ImmunoResearch Laboratories Inc. (West Grove, PA). Glibenclamide, glimepiride, gliclazide and diazoxide (DZ, a K_ATP_ channel opener) were all purchased from Sigma (St. Louis. MO, U.S.A). They were dissolved in dimethyl sulfoxide (DMSO) and stored at − 80 °C until use. Solutions of SUs as well as DZ were prepared fresh each day. Controls were performed in the presence of appropriate concentration of solvent (DMSO). Unless indicated, other reagents were from Sigma (St. Louis. MO).

To investigate the effects of the three SUs in HG-induced tubular epithelial cell apoptosis, the HK-2 cells were treated with these SUs for 24 h prior to exposure to 30 mM glucose for 24 h. To further investigate the role of K_ATP_ channels in this process, the HK-2 cells were treated with 100uM DZ for 24 h prior to exposure to SUs and 30 mM glucose.

### Cell viability assay

The HK-2 cells were seeded in 96-well plates at a concentration of 5 × 10^3^ Cells/ml and incubated at 37 °C. CCK-8 assay was employed to assess the viability of the cells. After being subjected to the above-mentioned treatments, the cells were washed with phosphate-buffered saline (PBS), and 10ul CCK-8 solution at 10% dilution was added to each well, and the plate was then incubated for approximately 24 h in an incubator. The absorbance at 450 nm was assayed using a microplate reader (Molecular Devices, Sunnyvale, CA, USA). The mean of the optical density (OD) of 3 wells in the indicated groups were used to calculate the percentage of cell viability according to the following formula: cell viability (%) = (OD_treatment group_/OD_control group_) × 100. The experiment was repeated 5 times.

### Apoptosis determination

The cells were incubated with 5 μl of Annexin V and 5 μl of propidium iodide (PI) for 15 min at room temperature in dark, according to the manufacturer’s instruction (BD Biosciences, SanJose, CA), and then subjected to flow cytometry to measure the apoptosis rate (%).

### Western blot analysis

Protein concentrations in cell extracts were determined (BioRad, Richmond, California, USA). Equal amounts of protein fractions of lysates were resolved over SDS-PAGE gels, transferred to PVDF membranes. After blocking with skim milk, membranes were incubated with anti-LC3I, LC3II, Bcl-2 and Bax. Corresponding secondary antibody were used. The peroxidase activity was detected by chemiluminescence using the ECL detection system. Optical density of the bands was quantified by densitometric analysis using ImageJ software (National Institutes of Health, USA). β-actin (#3700, Cell Signaling Technology) was used as an internal control.

### Statistical analysis

All statistical analyses were performed using Statistical Product and Service Solutions (SPSS) 19.0 software (from IBM). A Student’s t-test was used to assess significance for data within two groups. All data are presented as the means ± SEM, and significance was set at *P* < 0.05.

## Results

### Effect of SUs on human proximal tubule epithelial cell viability

Our results revealed that SUs exposure led to a dose-dependent inhibition of cell viability of the HK-2 cells by CCK-8 method. HK-2 cells treated with glibenclamide at a concentration of 45umol/L, gliclazide 1058umol/L, glimepiride 130 umol/L did not significantly affect cell viability. So, the above concentrations were used for our following experiments.

### Pro-apoptotic effect of glibenclamide on PTECs was alleviated, as well as an attenuated gliclazide protection, were seen with K_ATP_ channel opening

**Fig. 1:**
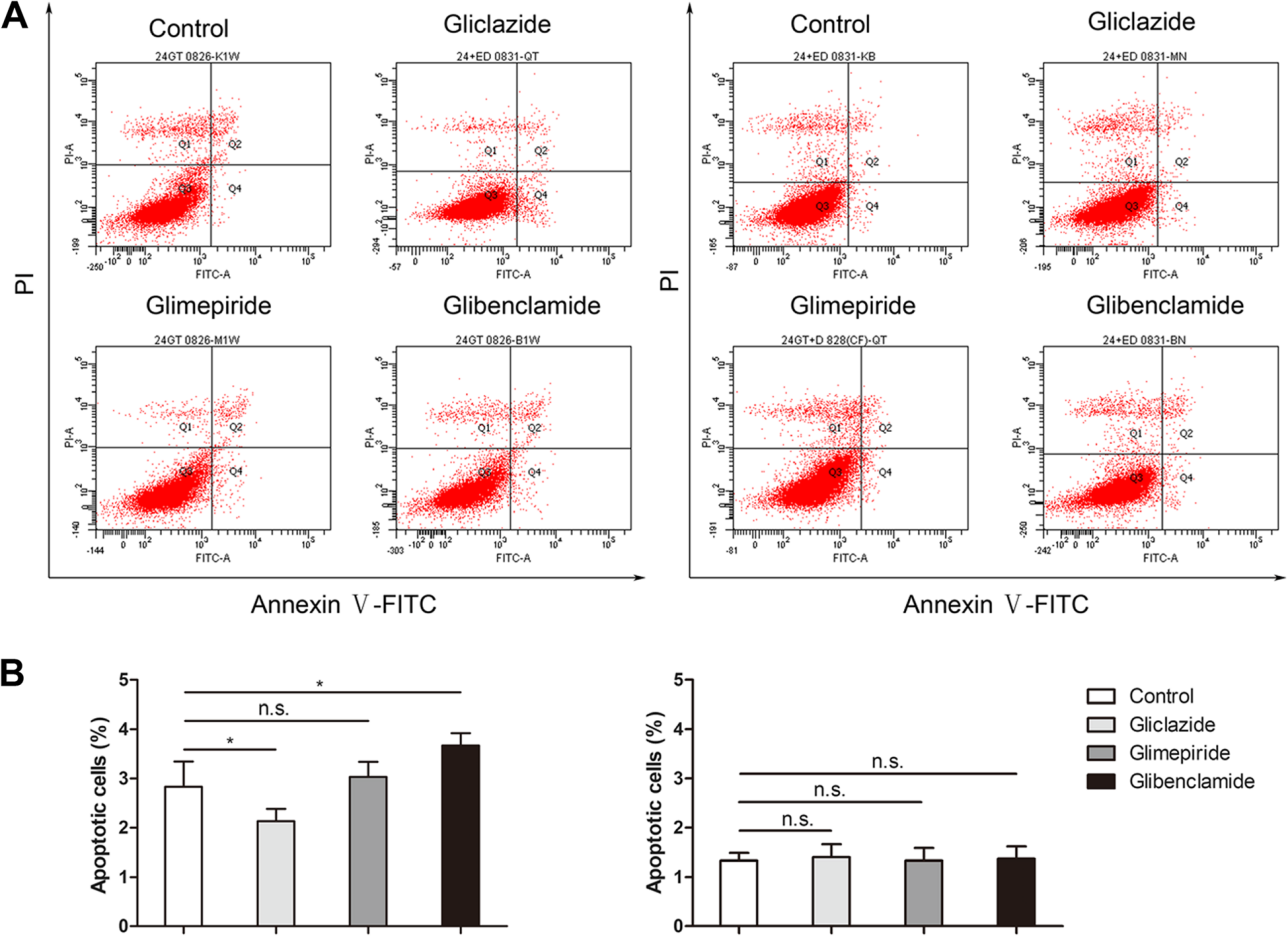
Effect of diazoxide on SUs-induced apoptosis was determined with Annexin V-FITC/PI staining by flow cytometry. **a** Flow cytometry results with Annexin V-FITC/PI staining. Cells were treated with SUs (left) and K_ATP_ channel opener (right), DZ, as described above. After culture for 24 h, cells were harvested and then apoptosis was analyzed with an Annexin V-FITC Apoptosis Detection Kit by flow cytometry. Cells were classified as healthy cells (Annexin V^−^, PI^−^), early apoptotic cells (Annexin V^+^, PI^−^), late apoptotic cells (Annexin V^+^, PI^+^), and damaged cells (Annexin V^−^, PI^+^). **b** The ratio of apoptosis among different experiment groups. Apoptosis ratio was early apoptosis percentage plus late apoptosis percentage. The date were presented as the mean ± SD. Columns, mean of three independent experiments; bars, SD; * *p <* 0.05, ** p < 0.01*,* n.s. not significant

To investigate the role of K_ATP_ channels in SUs-induced PTECs injury, the HK-2 cells were treated with or without 100uM of the putative K_ATP_ channel opener, DZ, for 24 h. Annexin-V binding and propidium iodide staining were used to detect apoptotic changes in HK-2 cells. As shown in Fig. 1, glibenclamide promoted the apoptosis of PTECs significantly (*P* < 0.05); the apoptosis rate (Q2 + Q4) in glimepiride-treated group was not different from the control group(*P* > 0.05). However, the effect was opposite in gliclazide group, where the apoptosis rate was reduced (*P* < 0.05). What’s more, the above effects of glibenclamide and gliclazide were alleviated by DZ. In contrast, exposure to glimepiride with or without DZ did not induce a significant change in the number of apoptotic cells.

**Fig. 2:**
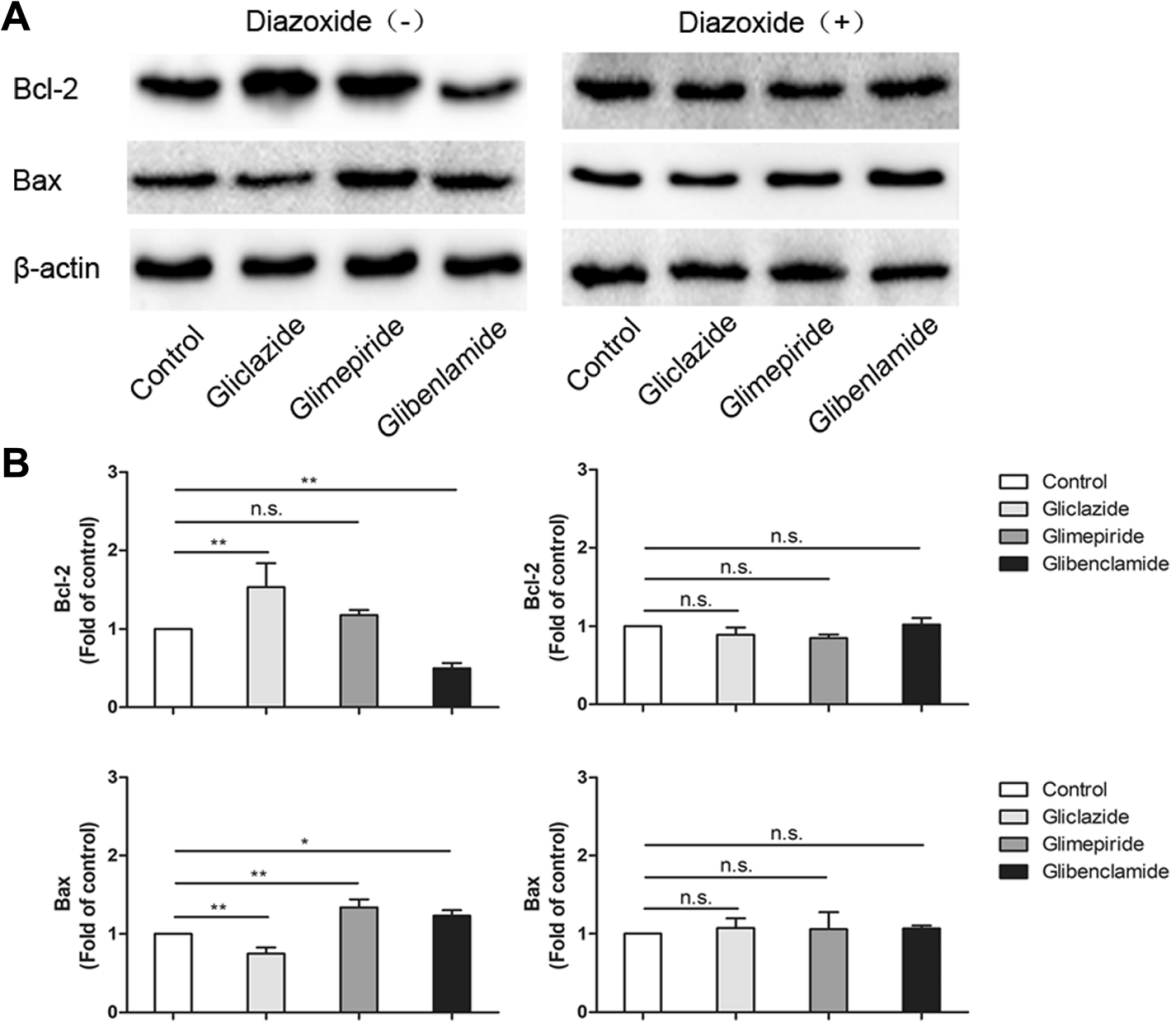
Bcl-2 and Bax expression in SUs and SUs + DZ groups. **a** Representative image of Western Blot of cells extracts. **b** Quantificaton of Western Blot results. Mean ± SD of three independent experiments *P < 0.05 versus control, ** p < 0.01, n.s. not significant

Bcl-2 is a crucial inhibitor and Bax is the promoter of apoptosis. Our results of western blotting showed that Bcl-2 was downregulated significantly (*P* < 0.01) and Bax was upregulated (*P* < 0.05) in glibenclamide group compared with that of control group (Fig. 2). The expression of Bax was upregulated significantly in glimepiride group compared to control group (*P* < 0.01), even though there was no significant difference in Bcl-2 expression. However, the results were opposite in gliclazide group, the expression of Bcl-2 was upregulated and Bax was downregulated significantly (all *P* < 0.01). The effects of glibenclamide, glimepiride and gliclazide on apoptosis were restored or even reversed by DZ, a putative K_ATP_ channel opener.

### The decreased autophagy-related protein and ratio of LC3-II/LC3-I were reversed by opening K_ATP_ channel

**Fig. 3:**
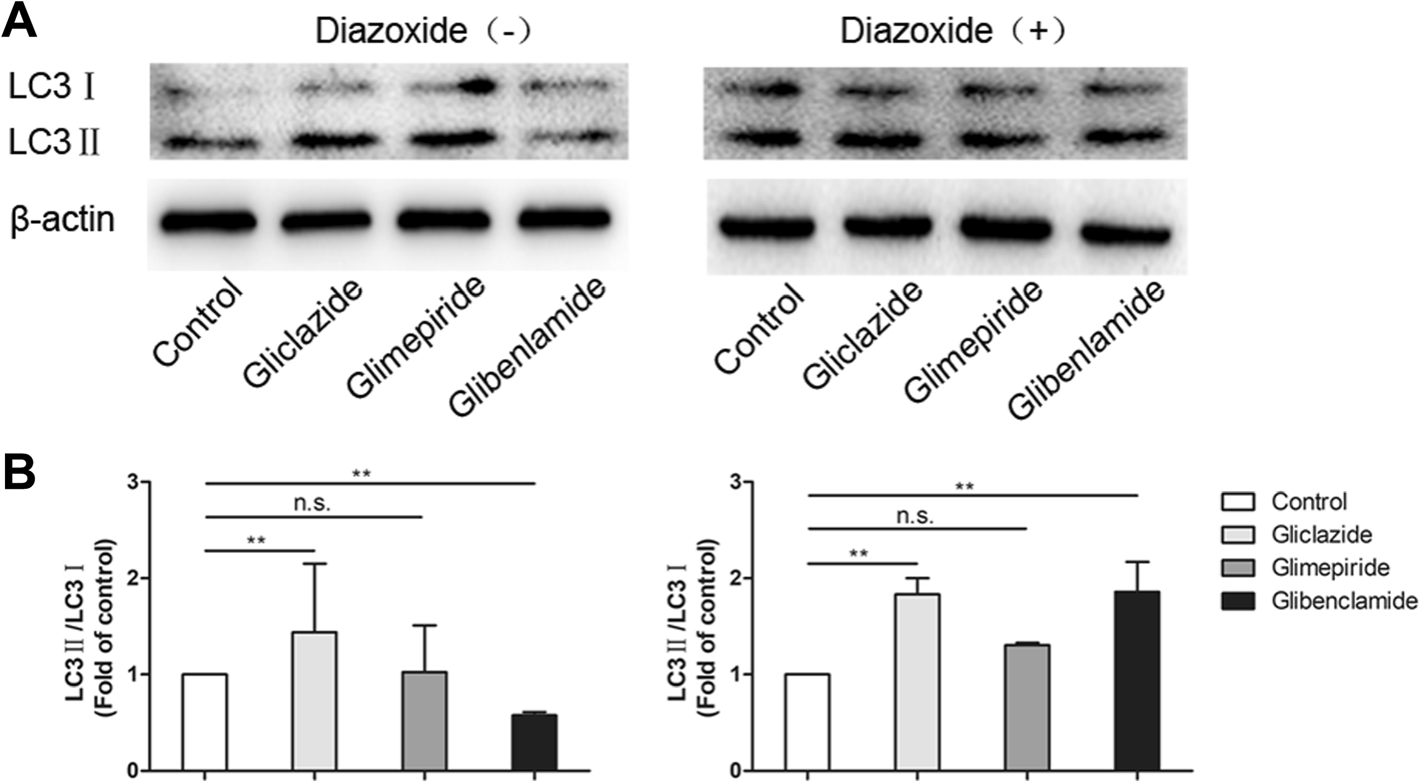
. Effect of SUs and SUs + DZ on expression of autophagy-related protein. LC3I and LC3II expression in SUs and SUs + DZ groups. **a** Western Blot analysis of cells extracts confirmed almost complete loss of LC3I to LC3II conversion after treated with DZ. **b** Quantificaton of Western Blot results. Mean ± SD of three independent experiments * *P* < 0.05 versus control, ** p < 0.01, n.s. not significant

Apoptosis and autophagy are two important cellular processes with complex and interacting protein networks. The mechanisms linking autophagy and apoptosis are not fully defined, however, our previous study found that there was a negative correlation between apoptosis and autophagy in PTECs (Zhang et al. 2017). A high level of constitutive basal autophagy is observed in PTECs, which is proved an indispensable process in this part of kidney (Isaka et al. 2011; Weide & Huber, 2011). When autophagy is initiated, LC3 is processed from LC3-I to LC3-II. The increase of LC3-II and/or the ratio of LC3-II/LC3-I can be an indicator of the activation of autophagy to some degrees (Dancourt & Melia, 2014). As shown in Fig. 3, the expression of LC3-II and the ratio of LC3-II/LC3-I were increased in gliclazide and decreased in glibenclamide group significantly as compared with control (*P* < 0.01, *P* < 0.01). While their expressions did not show apparent difference in glimepiride group (*P* > 0.05). DZ upregulated the expressions of above two indicators more remarkable in glibenclamide than that in glimepiride group as compared with control group. These reversal changes were not found in gliclazide-treated HK-2 cells with or without DZ. The above results indicate that different SUs might have different effects on autophagy, and the changes of autophagy-related protein in glibenclamide can be reversed by opening the K_ATP_ channel.

## Discussion

Increasing evidences suggest that apoptosis of tubular epithelial cells, especially PTECs, play an important role in the progression of DKD (Wakino et al. 2015; De Nicola et al. 2014; Singh et al. 2008). Sulfonylureas have been reported to accelerate apoptosis and dysfunction of pancreatic beta cells due to sustained enhancement of Ca^2+^ influx and stimulated production of reactive oxygen species (ROS) (Efanova et al. 1998; Iwakura et al. 2000; Tsubouchi et al. 2005). Although SUs have long been utilized for their hypoglycemic properties in DKD patients, little evidence has been reported about their influences on the progression of DKD. The purpose of our study was to explore the effects and possible mechanisms of three widely prescribed SUs on the apoptosis of PTECs. The results showed that glibenclamide increased apoptosis of PTECs significantly, whereas the apoptosis of PTECs treated with gliclazide was reduced. Although glimepiride did not significantly increase the apoptosis rate of PTECs, the expression of proapoptotic protein Bax was increased in this group. Moreover, the increased apoptosis induced by glibenclamide, as well as the increase of Bax expression caused by glimepiride, could be alleviated or even restored obviously by K_ATP_ channel opener, implying that closure of the K_ATP_ channel might contribute to the apoptosis induced by specific SUs.

Why different SUs have different effects on apoptosis of PTECs? The K_ATP_ channels are hetero-octameric complexs of pore-forming inwardly rectifier K^+^ (Kir6) channel-forming subunits associated with regulatory SUR subunits (Inagaki et al. 1995). Two Kir6-encoding genes, KCNJ8 (Kir6.1) and KCNJ11 (Kir6.2), and 2 SUR genes, ABCC8 (SUR1) and ABCC9 (SUR2), encode mammalian K_ATP_ subunits, but alternative RNA splicing can cause multiple SUR protein variants(e.g. SUR2A and SUR2B) that confer distinct physiological and pharmacological properties on the channel complex (Inagaki et al. 1996; Chutkow et al. 1996; Babenko et al. 2000). SUR endow the channel with sensitivity to SUs (Tucker et al. 1997). In addition to stimulating the insulin secretion by binding with SUR1 on the membrane of islet beta cell, SUs could also bound to its specific SURs in various other extrapancreatic tissues in the body, causing K_ATP_ channel closure (Tsubouchi et al. 2005; Babenko et al. 2000; Liu et al. 2016). It has been estimated that cardiac and skeletal muscle channels contain Kir6.2 and SUR2A, and smooth muscle K_ATP_ channel are formed by the coupling of SUR2B with either Kir6.2 or Kir6.1 (Liu et al. 2015). Either glibenclamide or glimepiride could inhibit both Kir6.2/SUR1 and Kir6.2/SUR2A currents with high affinity, promoting closure of the K_ATP_ channel and reducing ischemic preconditioning (Thompson et al. 2013). SUR2B is widely expressed in the kidney, particularly in proximal tubule, ascending limb, and the collecting duct, where it presumably mediates, in part, K^+^ transport (Chutkow et al. 2002). Evidence suggests that the effect of SUs on these K_ATP_ channels in different tissues varies. Considering the different effects of the three SUs on apoptosis in HK-2 cells, several possible mechanisms can be proposed.

First, conventional and modern SUs may display different sensitivity and specificity towards K_ATP_ channels on PTECs. The binding of glibenclamide with K_ATP_ channels is unselective and hardly reversible (Chutkow et al. 2001). Although the selectivity of glimepiride with K_ATP_ channels is similar in SUR1 (in pancreas) and SUR2 (extrapancreatic tissues), but it dissociates quickly from the binding site and its blockage is reversible (Kakkar et al. 2006). On the other side, gliclazide shares with glibenclamide and glimepiride a sulphonylurea moiety but does not possess a carbox-amido-ethyl-phenyl group attached to the phenyl-sulphonyl group, which in part explains its specific interaction with the SUR1 and producing a reversible inhibition of K_ATP_ channels (Ashcroft & Gribble, 2000). Such different binding selectivity and reversibility of these SUs may have produced different interactions in apoptosis of PTECs.

Second, several reports have shown that oxidative stress play an important role in the impairment of renal tubular function. Glibenclamide and glimepiride were reported to stimulate ROS production in the pancreatic beta cell line MIN6 (Tsubouchi et al. 2005). The increased ROS production is a causative mechanism for the apoptosis of β-cells induced by them. In contrast, gliclazide did not significantly stimulate ROS production. Beyond that, gliclazide, a modern SUs with a bicycle-octyl ring structure, scavenged ROS, inhibited NADPH oxidase and glomerular macrophage infiltration with suppression of ICAM-1, and prevented renal damage (Onozato et al. 2004). Similar effects might exist as the potentially protective mechanism of gliclazide that is different from other SUs.

Third, mechanisms other than K_ATP_ channel are involved in PTEC apoptosis (Gribble et al. 1998). We could not exclude the possibility that these factors play a role in the process of apoptosis induced by specific SUs.

These evidences suggest that glibenclamide and glimepiride, especial the former, might promote the apoptosis of PTECs through closing the K_ATP_ channel by binding to the SUR.

What might be the possible mechanisms of SUs induced apoptosis of PTECs by closing K_ATP_ channel? It has been reported that SUs can induce apoptosis in β-cells or clonal β-cell lines under certain conditions, however, the mechanism is still unidentified (Beesley et al. 1999). Kim et al. (Kim et al. 2012) reported that SUs could induces apoptosis not only by elevated cytosolic Ca^2+^ but also by dysregulation of Ca^2+^ homeostasis through interfere with the endoplasmic reticulum stress (ERS). ERS is involved in a number of physiological and pathological processes, including diabetes. The ability of renal tubular cells to cope with ERS is essential for maintaining normal renal function (Ashcroft, 2006). Recent research suggests that ERS is a major factor in renal tubular cell apoptosis resulting from ischemic acute kidney injury (Sliwinska et al. 2012). Therefore, SUs’ closure of K_ATP_ channels, increased intracellular Ca^2+^, which might have caused the ERS and triggered the apoptosis of PTECs. Apoptosis induction by specific SUs depends on different SUR isoform expression in different tissues. SUR2B was reported to express in PTECs, which may be regarded as the main target of SUs, responsible for mediating the process.

Autophagy is a highly conserved cytoprotective process, which allows cells to mitigate various types of cellular stress (Kim et al. 2012). Several groups have shown that autophagy plays a critical role in maintaining tubule homeostasis and integrity under conditions of stress (Inoue et al. 2010; Periyasamy-Thandavan et al. 2008; Yang et al. 2008; Jiang et al. 2010). More importantly, Shuya Liu et al. (Liu et al. 2012) found that proximal tubule cells depend more than any other tubule segment on basal autophagic activity. Our previous study also showed that impairment of autophagy play an important role in high glucose (HG)-induced apoptosis of PTECs. Whereas, the upregulation of autophagy in HK-2 cells could protect against HG-mediated apoptosis (Zhang et al. 2017). Here, our results suggested that impairment of autophagy induced by SUs might also contribute to the apoptosis of PTECs. In addition, our results are consistent with previous observations that autophagy protects PTECs from injury and apoptosis (Dong et al. 2015; Xu et al. 2016). Other research indicates that increased cytosolic Ca^2+^ levels regulated by K_ATP_ channels play a role in impairing autophagy, which is associated with neurodegenerative disorders (Hambrock et al. 2006). Altogether these studies brought an assumption that SUs could interfere with autophagy through their action on K_ATP_ channels, which then play a part in SUs-induced apoptosis of PTECs.

Over the past decades, although several studies focused on the hypoglycemic effects of SUs on DKD treatment, few studies paid attention to the direct effect of SUs on kidney, especially their effects on the apoptosis of PTECs. Emerging evidences showed that K_ATP_ channels are involved in regulating energy metabolism and maintaining the homeostasis in podocyte and renal tubular cells (Kim et al. 1999; Meijer & Codogno, 2008; Havasi & Dong, 2016). Our study finds that different SUs induce the apoptosis of proximal tubular epithelial cell differently. Glibenclamide and glimepiride, in particular the former may promote the apoptosis of PTECs; those effects may be reversed by DZ. Gliclazide, on the other hand, decreased the apoptosis of PTECs. Calcium overload induced by closure of K_ATP_ channels might contribute to the impairment of autophagy and subsequent activation of apoptosis. Further precise mechanisms remain to be determined.

There are several limitations in this study, first of which being lack of a *SUR2* gene knockdown group. Second, the autophagy activity in tubular cells has not yet been elucidated in detail in this study. Third, only three common SUs included in our study, whether our findings could be generalized to other SUs need to be examined in further study.

## Conclusions

In summary, our study provides evidence for the first time that SUs, such as glibenclamide, could induce apoptosis in PTECs through a K_ATP_ channel-dependent manner and this effect could be reversed by DZ. Our data also indicated that different SUs induce apoptosis differently in PTECs. SUs should not be regarded as a homogeneous drug class in terms of their tissue specificity and their effects on extra-pancreatic cells. The clinical relevance of extra-pancreatic action of SUs is being widely discussed and remains controversial. Although it is still uncertain if SUs are harmful to renal cells, a more appropriate strategy for diabetes treatment would be to use drugs acting specifically on the β-cell K_ATP_ channels.

## Abbreviations

DKD: Diabetic kidney disease
DMSO: Dimethyl sulfoxide
DZ: Diazoxide
ERS: Endoplasmic reticulum stress
HG: High glucose
K_ATP_: Channel: ATP-dependent potassium channel
PTECs: Proximal tubular epithelial cells
ROS: Reactive oxygen species
SURs: Sulfonylurea receptors
SUs: Sulfonylureas
T2DM: Type 2 diabetes mellitus
